# Restless Legs and Iron Deficiency: Unraveling the Hidden Link and Unlocking Relief

**DOI:** 10.7759/cureus.82413

**Published:** 2025-04-17

**Authors:** Fatema M Alzaabi, Dana J Al Tarawneh, Yusuf J Al Tarawneh, Abdallah Khan, Mohammed Abdul Muqsit Khan, Tabish W Siddiqui, Raqshan W Siddiqui, Syed Muhammad Hayyan Nishat, Asma A Alzaabi, Shiza W Siddiqui

**Affiliations:** 1 Internal Medicine, RAK Medical and Health Sciences University, Ras Al Khaimah, ARE; 2 Internal Medicine, King Khalid University Hospital, Abha, SAU; 3 Research, Dubai Medical College, Dubai, ARE

**Keywords:** iron-deficiency, iron markers, iron supplementation, restless legs syndrome, rls

## Abstract

Restless legs syndrome (RLS), also called Willis-Ekbom disease (WED), is a condition marked by an overwhelming urge to move the legs, particularly during rest or at night. Treatments, including intravenous iron, show potential, but further studies are needed to establish their long-term safety and efficacy. The findings integrate various studies to better understand diagnosis, mechanisms, and treatment options for RLS. This review explores the connection between RLS and iron deficiency, emphasizing iron metabolism and its potential role in disease development.

## Introduction and background

An insatiable drive to move is the hallmark of restless legs syndrome (RLS), sometimes referred to as Willis-Ekbom disease (WED) [1]. This prevalent neurological movement disorder primarily affects the legs, typically later in the day, and worsens at night [1,2]. Researchers estimate that 7% of people in five European nations suffer from RLS [1,2]. The illness is commonly overlooked due to the frequency of sensory symptoms involving the lower extremities [2]. The defining feature of RLS, a circadian dysfunction of sensorimotor integration, has given rise to several hypotheses about the pathophysiological processes underlying the condition [2]. 

Our clinical understanding of RLS has improved due to our increased knowledge of the disorder's etiopathogenesis and examination of both endogenous and exogenous factors, such as central nervous system (CNS) dopaminergic and iron deficiency (ID) hypotheses, as the etiologic hallmarks of RLS [2,3]. RLS often runs in families, with 83% of monozygotic twins concordant [4]. The inheritance pattern is usually autosomal dominant, primarily when the onset occurs at a young age [5]. Genome-wide association studies have found at least eight implicated loci [6]. Dopaminergic dysfunction plays a crucial role in RLS, but it is not solely due to CNS dopaminergic deficiency, even though dopaminergic agents and dopamine agonists improve symptoms [7]. Since Ekbom's early descriptions, ID has been linked to RLS, focusing on brain iron rather than serum ID, which is present in only 25% to 44% of patients with RLS [8].

Many observational studies and clinical trials have examined the relationship between ID and RLS, and studies have found that low-serum ferritin levels significantly correlate with the prevalence or severity of RLS [9,10]. One study showed that, although hemoglobin and serum ferritin levels (both within the normal range) did not differ between the RLS and control groups, RLS subjects had significantly higher cerebrospinal fluid transferrin and significantly lower ferritin, both consistent with CNS ID [11]. Although serum iron is regularly measured along with a more detailed set of tests, including ferritin, transferrin, and iron-binding capacity, the critical factor in RLS is the iron level in the synaptic cleft, as this directly correlates with RLS symptoms [12,13].

This study evaluates the relationship between RLS and ID, focusing on how iron levels influence the condition and examining the impact of iron supplementation on these symptoms. It also aims to deepen our understanding of RLS by examining its underlying pathophysiology and evaluating current management strategies. This review seeks to provide valuable insights into improving the diagnosis and treatment of RLS, ultimately enhancing patient care.

## Review

Methodology

Search Strategy

**Figure 1:**
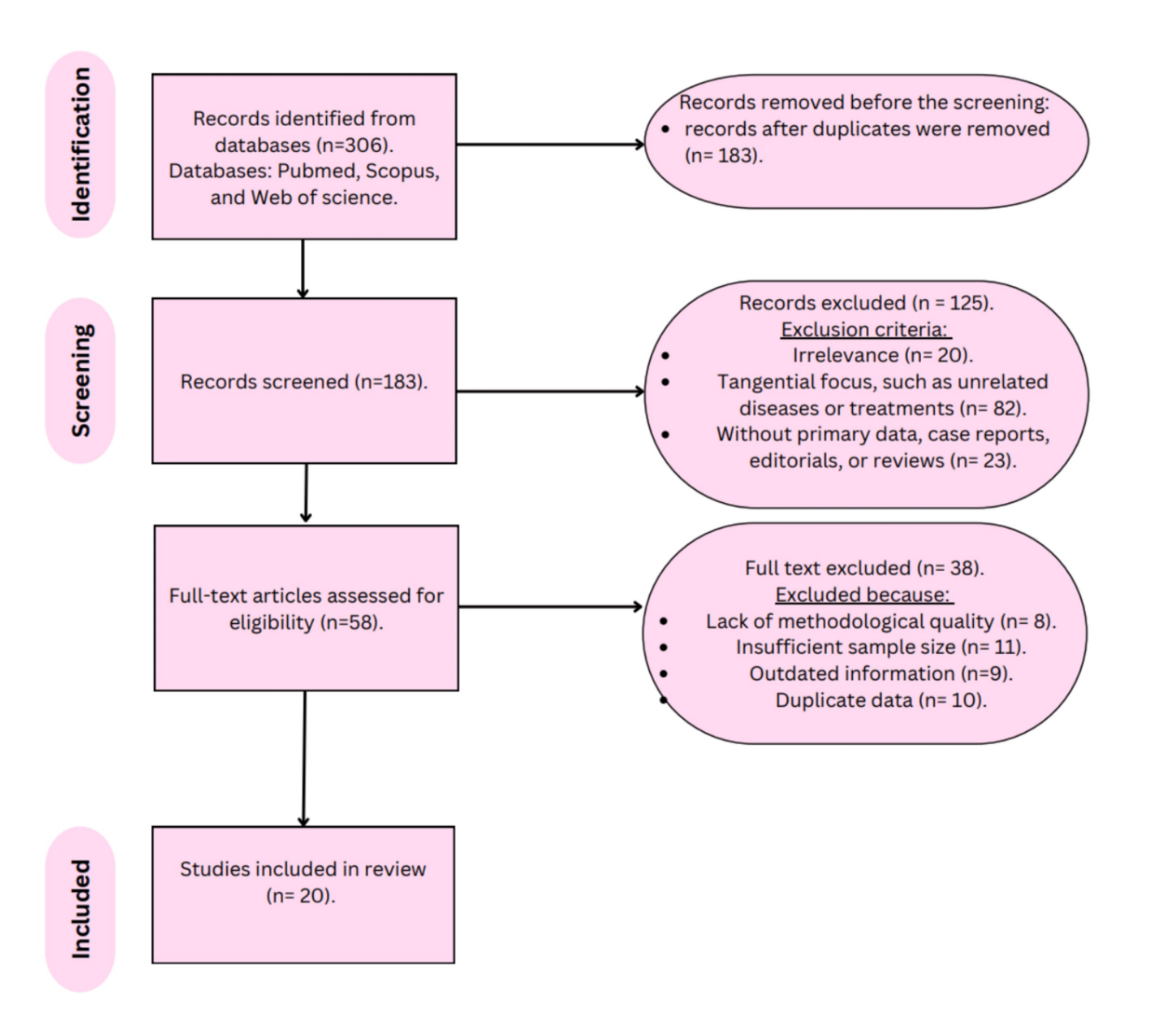
PRISMA flowchart of selected articles. Image credit: Fatema Marwan Alzaabi. PRISMA, Preferred Reporting Items for Systematic Reviews and Meta-analyses

This systematic review followed the Preferred Reporting Items for Systematic Reviews and Meta-analyses (PRISMA) standards. A thorough search was undertaken in the PubMed, Scopus, and Web of Science databases using a mix of keywords such as "iron deficiency," "restless legs syndrome," "RLS," "iron markers," and "iron supplementation." Boolean operations like AND and OR generated sophisticated search strings, ensuring sensitivity and specificity in discovering relevant research. For example, the search phrases included "iron deficiency," "restless legs syndrome," and "iron markers" OR "iron supplementation" AND "RLS." The search was limited to English-language studies, available as free full-text publications with no publication date restrictions. The last search was conducted on January 23, 2025. Initial database queries returned 306 articles; after deleting duplicates, 183 records remained. Titles and abstracts were checked for relevance, resulting in 58 possibly qualifying papers. Following a full-text examination, 20 papers were selected for the final analysis. The PRISMA flow diagram depicts the study selection process (Figure 1).

Inclusion and Exclusion Criteria

The inclusion criteria focused on research published in English that investigated the link between ID and RLS or assessed iron-related indicators such as ferritin, transferrin saturation (TSAT), or serum iron levels. Eligible studies had to be available as free full-text publications and employ relevant study designs, such as cross-sectional, cohort, randomized controlled trials, or case-control methods. Studies were eliminated if they were not in English, did not provide free full-text access, or focused on topics unrelated to the aims of the review. During the full-text review, additional exclusions were made based on the following reasons: lack of statistical analysis or data interpretation, insufficient sample sizes that compromised the validity of the results, outdated information that no longer reflected current research trends, and duplication of data that overlapped with other included studies.

Results

The initial search retrieved 306 articles, with 183 remaining after duplicate removal. Screening of titles and abstracts excluded 125 records deemed irrelevant to the review objectives. Full-text reviews of the remaining 58 articles resulted in the exclusion of 38 studies. Reasons for exclusion included lack of statistical analysis, insufficient sample sizes, outdated findings, and data duplication. A total of 20 studies met the inclusion criteria and were included in the final synthesis. These studies represented a mix of study designs, including cross-sectional studies, cohort studies, randomized controlled trials, systematic reviews, meta-analyses, and case-control studies. Most studies reported that low serum ferritin levels and other markers of iron deficiency were typical in patients with RLS.

Furthermore, multiple studies identified the potential advantages of iron supplementation, particularly in people with low baseline iron levels. There was a bias toward positive-result articles, with repeated duplicate citations limiting diversity and breadth. The PRISMA flow diagram provides a complete overview of the research selection process (Figure 1).

Discussion

The relationship between RLS and ID has been a focus of research for decades, with significant attention paid to how iron dysregulation influences the pathophysiology of this movement disorder [1,2,3]. The hallmark of RLS, a strong urge to move the legs, often intensifies at night, causing significant distress and disruption in patients' lives [1]. Although much of the early understanding of RLS pointed to CNS dopaminergic dysfunction, iron deficiency, particularly within the brain, has emerged as a crucial factor in the disorder's development [2]. RLS is more commonly found in women than men, and its prevalence increases with age [14,15]. Among populations of Western European descent, RLS affects about 5% to 15%, whereas it is less common in other groups, such as specific Asian populations, where prevalence ranges from 0.1% to 5% [16].

Diagnosis and Severity Assessment of RLS

RLS is a common neurological condition that significantly influences sleep, health, and quality of life [17]. Genetics plays a significant role in RLS, as many affected individuals report a family history. Familial linkage studies have pinpointed several genetic regions linked to RLS [18]. A Genome-wide association study has further identified five genes - BTBD9, MEIS1, MAP2K5, PTPRD, and TOX3 - that contribute to an increased risk for RLS or associated periodic limb movements during sleep, accounting for about 80% of the population's genetic risk [19]. RLS diagnosis relies on meeting five essential criteria outlined by the International RLS Study Group (IRLSSG) in Table 1 [20].

**Table 1:** Five essential criteria for diagnosing RLS. Source: [20]. RLS, restless legs syndrome

International RLS Study Group (IRLSSG) diagnostic criteria
A need to move the legs is usually accompanied or caused by uncomfortable, unpleasant sensations in the legs.
Symptoms are exclusively present or worsen during times of inactivity/rest.
Partial or total relief of symptoms by movement, such as walking or stretching, at least as long as the activity continues.
Symptoms are generally worse or exclusively occur in the evening or during the night.
The occurrence of the first four essential criteria must not be solely accounted for as symptoms primary to another medical or behavioral condition.

For quick screening, the IRLSSG recommends a single validated question: When you try to relax in the evening or sleep at night, do you ever have unpleasant, restless feelings in your legs that can be relieved by walking or movement? This question has proven highly effective in large-scale screenings, with 100% sensitivity and 96.8% specificity for detecting RLS [21]. However, while this question helps identify potential RLS cases, a formal diagnosis requires a thorough review of the patient's history and symptoms to confirm alignment with the IRLSSG criteria and rule out any secondary conditions that may cause similar symptoms [22].

The IRLSSG rating scale is a validated disease-specific instrument for measuring RLS severity. It is considered the gold standard for evaluating outcomes in clinical trials [23].

Pathophysiological Mechanisms of RLS

ID has been implicated in the pathophysiology of RLS through various mechanisms, particularly its role in dopamine regulation and brain function [3]. Iron exists in ferric (Fe3+) and ferrous (Fe2+) forms, with ferrous iron being more reactive [24]. Iron is primarily bound to transferrin in the blood and stored in ferritin within cells, facilitating delivery to tissues, including the brain [25]. Hepcidin, a key regulator, limits iron absorption during inflammation or elevated iron levels, reducing the efficacy of oral iron supplements [26].

Research indicates that RLS may arise from brain ID, even when systemic iron levels appear normal [27]. Studies show reduced brain iron, particularly in the substantia nigra, impairing dopamine D2 receptor function [28]. This deficiency correlates with reduced cerebrospinal fluid (CSF) ferritin levels and increased transferrin, contributing to dopaminergic dysfunction and RLS symptom severity [29]. Dopamine’s central role in movement and sensory processing links these deficits to the disorder’s hallmark symptoms, which include a circadian activity pattern decreasing in the evening and night and increasing in the morning [30]. Furthermore, iron chelation studies suggest that the dopamine D2 receptor is iron-dependent, with deficiency leading to hypofunction [31]. Aging-related reductions in D2 receptor binding sites may also contribute to the higher prevalence of RLS in older adults [32,33]. These findings support that iron deficiency in the brain, particularly in dopamine-related pathways, is a critical factor in developing RLS [30].

Hypoxic-state activation is another proposed mechanism in RLS, with elevated hypoxia-inducible factors (1-alpha and 2-alpha) and vascular endothelial growth factor (VEGF) observed in the microvasculature of patients with RLS [34,35]. ID may disrupt oxygen transport, affecting iron regulation across the blood-brain barrier [36]. Hypoxemia in peripheral tissues has also been linked to symptom severity, with dopaminergic therapy partially reversing both hypoxemia and symptoms [37]. 

**Figure 2:**
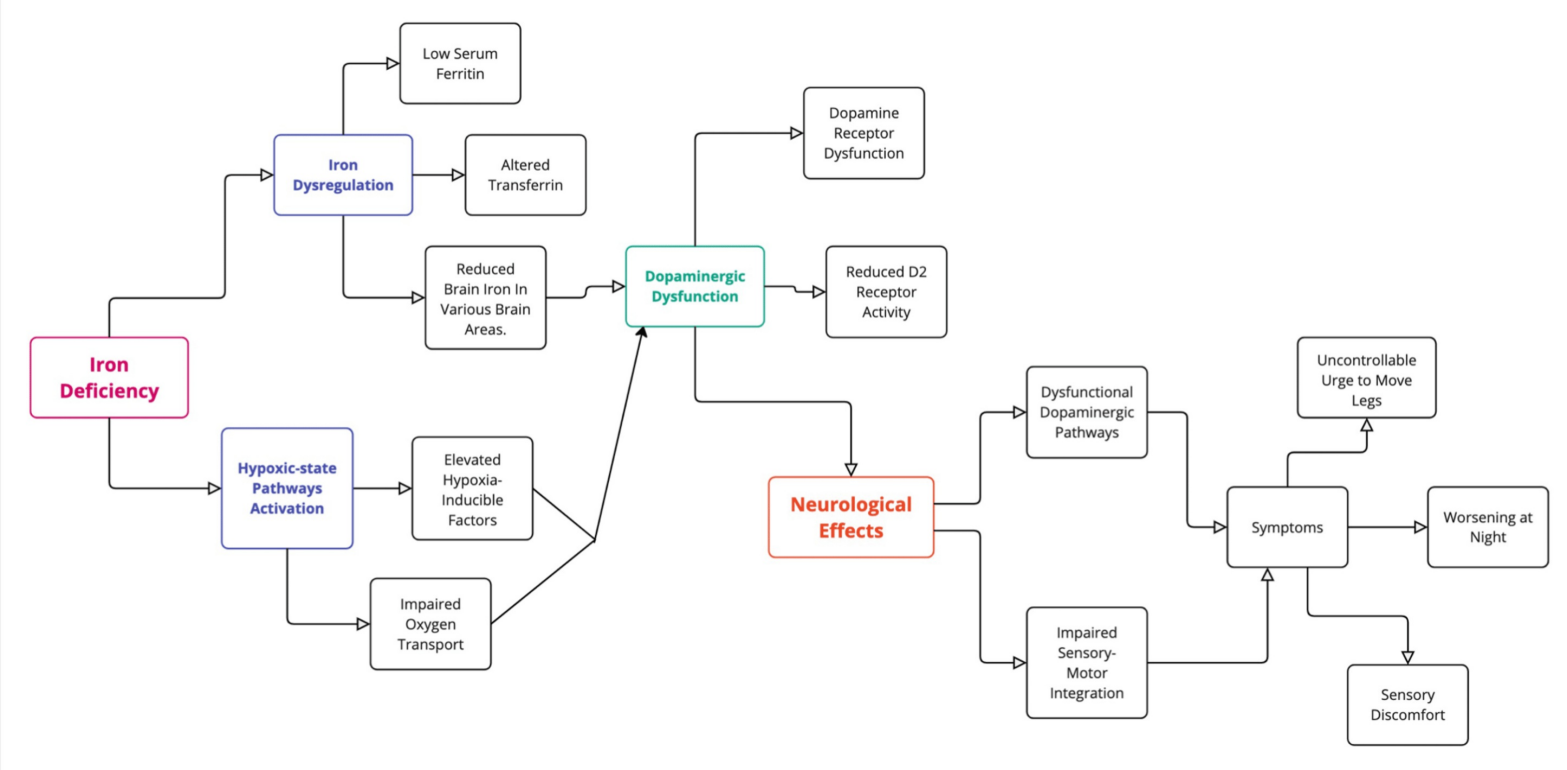
Pathophysiology of RLS. Image credit: Fatema Marwan Alzaabi. RLS, restless legs syndrome

Genetic factors and a hypo-adenosinergic state have also been implicated, with low adenosine levels promoting hyperarousal and further activating dopaminergic and hypoxic pathways [38,39]. Current evidence underscores systemic iron measures poorly reflect regional brain iron, highlighting the need for advanced tools to assess localized brain IDs [11]. The pathophysiology of RLS is illustrated in Figure 2, providing a visual overview of the underlying mechanisms involved.

Iron Dysregulation and Its Role in RLS

Iron regulation in the body is a complex, organ-specific process influenced by genetic factors and physiological demands [12]. Standard clinical measures of iron status, such as TSAT, ferritin, and hemoglobin, reflect iron availability for erythropoiesis but do not provide insights into iron levels in organs like the brain, where regulation occurs independently [40].

The link between ID and RLS was first identified by Ekbom in 1960 [8]. Later, Sun et al. confirmed that patients with RLS had significantly lower serum ferritin levels than controls, establishing a connection between systemic iron deficiency and symptom severity [10]. Earley et al. demonstrated central ID in RLS through CSF analysis, showing lower ferritin and higher transferrin concentrations in patients, indicative of impaired iron transport across the blood-brain barrier [11]. The cerebral capillary transferrin receptor, crucial for regulating iron at the blood-CSF interface, is key in maintaining brain iron levels [13]. Additionally, RLS symptoms follow a circadian pattern, worsening at night. Serum iron also exhibits a marked circadian variation, with concentrations dropping by 30%-50% at night [41]. These findings suggest CSF ferritin and transferrin as promising biomarkers for RLS diagnosis and management [11].

In pediatric populations, ID is also implicated in RLS. Cielo et al. reported a higher prevalence of RLS symptoms and periodic limb movements among children born prematurely, a group at high risk of ID due to limited prenatal iron stores [42]. Premature infants are particularly vulnerable to iron deficiency; up to 85% of infants born at less than 32 weeks gestation and with a birth weight <1,500 g have iron deficiency during infancy [43]. Iron is particularly important in the development of the fetal brain, which grows rapidly during the first two years of life. In addition to impacting areas of the brain involved in learning, memory, and cognition, iron is an essential co-factor in synthesizing neurotransmitters like dopamine [44]. A study of 32 children (aged 10 years) with a prior diagnosis of iron deficiency during infancy revealed an increased periodic limb movement index (PLMI) during overnight polysomnography compared to controls [45]. This may contribute to early-onset RLS and sleep-related movement disorders in this population [42].

Bae et al. documented a high prevalence (40.3%) of RLS among patients with ID in Korea [46]. Similar patterns have been observed in Europe [9] and the United States [47], where RLS prevalence is higher among ID patients. In the same study, 33.1% of ID patients reported severe to very severe RLS, slightly exceeding rates observed in Western populations [46]. Advanced imaging techniques, such as magnetic resonance imaging (MRI), further support the role of central ID in RLS [11,29,47]. Reduced iron concentrations in brain regions like the substantia nigra and putamen have been observed in patients with RLS, emphasizing brain ID as a core feature of the condition [11,29,47].

Association of Iron Deficiency and RLS

The studies in Table 2 show a substantial link between low ferritin levels and the severity of RLS. O'Keeffe et al. found that older patients with RLS with lower ferritin levels had more severe symptoms, highlighting the importance of ID (with or without anemia) in RLS development among the elderly (*P* < 0.05) [9]. Similarly, Sun et al. discovered a link between low ferritin levels and worsening RLS symptoms; however, their study did not look at supplementation [10]. Both studies use serum ferritin as a crucial measure, but only O'Keeffe et al. investigated the effects of oral iron treatment and found it beneficial for relieving symptoms [9,10]. These findings highlight the importance of ferritin in determining RLS severity, particularly in elderly populations [9,10].

**Table 2:** Association of iron deficiency and restless legs syndrome. CSF, cerebrospinal fluid; RLS, restless legs syndrome; IRLSSG, International Restless Legs Syndrome Study Group; PSG, polysomnography; FCM, ferric carboxymaltose; TSAT, transferrin saturation; ICSD-3, International Classification of Sleep Disorders, 3rd edn.; PLMD, periodic limb movement disorder; IV, intravenous; LMWID, low-molecular-weight iron dextran; HMWID, high-molecular-weight iron dextran; IDA, iron deficiency anemia

Study	Sample size	Type of study	Population characteristics	Iron markers assessed	RLS severity measure	Main outcome	Iron supplementation	*P*- value
O'Keeffe et al. [9]	18	Case-control	Elderly patients with RLS	Serum ferritin, serum iron, vitamin B12, folate, and hemoglobin	IRLSSG Rating Scale [50]	Lower ferritin levels were associated with more severe RLS symptoms. ID, with or without anemia, significantly affects RLS development among elderly patients.	Oral iron (ferrous sulfate)	<0.05
Sun et al. [10]	27	Retrospective observational with blinded procedures	Adult patients (aged 29-81 years) with RLS	Serum ferritin	Clinical ratings and PSG measures	Lower ferritin levels correlated with greater RLS severity and reduced sleep efficiency	Not studied	N/A
Earley et al. [11]	24	Observational	16 RLS patients, 8 healthy individuals	CSF ferritin and transferrin, serum iron, ferritin, and transferrin	Clinical ratings and PSG measures	RLS patients had significantly lower CSF ferritin and higher CSF transferrin levels compared to controls, while no significant differences were observed in serum markers.	Not studied	<0.001
Trenkwalder et al. [27]	110	Randomized clinical trial	Patients with RLS nonanemic but iron-deficient	Serum ferritin, TSAT	IRLSSG Rating Scale [50]	FCM treatment significantly increased serum ferritin and TSAT levels compared to placebo. No correlation was found between baseline serum ferritin levels or changes in iron parameters and improvements in RLS symptoms.	IV FCM	<0.001
Cielo et al. [42]	167	Observational	Children aged 5-12 years with a history of prematurity	Serum ferritin	ICSD-3 and PSG measures [51]	Higher prevalence of RLS in preterm children	Not studied	<0.001
Allen et al. [12]	31	Systematic review/guideline formulation	Adults and children with RLS or PLMD	Serum ferritin, TSAT	IRLSSG Rating Scale [50]	Oral iron is the first-line treatment. IV iron should be considered when serum ferritin levels are too high for effective oral absorption or when oral iron is not tolerated or contraindicated.	Oral options include ferrous sulfate combined with vitamin C, while IV options include FCM, iron sucrose, low molecular weight iron dextran (LMWID), iron gluconate, high molecular weight iron dextran (HMWID), ferumoxytol, and iron isomaltoside.	N/A
Rosen et al. [49]	47	Cohort	Pediatric patients aged 5-18 years with RLS	Serum ferritin	Pediatric IRLSSG Rating Scale [52]	A modest, yet nonsignificant, improvement was observed in children with RLS symptoms, despite a significant increase in ferritin levels.	Oral iron (ferrous sulfate + vitamin C)	>0.05
Macher et al. (2020) [48]	176	Randomized clinical trial	Iron-deficient blood donors	Ferritin, TSAT, hemoglobin	IRLSSG Rating Scale [50]	A significant improvement in RLS symptoms between baseline and after IV or oral iron was seen. IV iron is more effective than oral.	IV (FCM) vs. oral iron (iron fumarate)	<0.001
Bae et al. [46]	124	Observational cohort study with a case-control comparison	Adults with IDA and RLS	Ferritin, TSAT, hemoglobin, iron, total iron binding capacity	IRLSSG Rating Scale [50]	There is a high prevalence of RLS among patients with IDA, with the majority displaying severe to very severe symptoms.	Not studied	<0.001

Trenkwalder et al. and Macher et al. conducted randomized controlled studies to examine the efficacy of intravenous (IV) versus oral iron supplementation for RLS therapy [27,48]. Trenkwalder et al. found that ferric carboxymaltose (FCM) increased serum ferritin and TSAT levels, although there was no clear correlation between baseline ferritin and RLS improvement (*P* < 0.001) [27]. Macher et al. found that IV iron treatment significantly improved symptoms compared to oral formulations, especially for severe iron shortage (*P* < 0.001) [48]. While both studies indicate IV iron as a better strategy, Macher et al.'s findings highlight its potential for long-term symptom management, particularly for patients who do not respond to oral iron [27,48].

Studies by Earley et al. [11] and Bae et al. [46] provide complementary information on the effect of iron on RLS. Earley et al. studied CSF indicators and found that patients with RLS had considerably lower CSF ferritin and greater CSF transferrin than controls, indicating central ID as a leading cause of the condition (*P* < 0.001) [11]. In contrast, Bae et al. examined a more extensive range of serum indicators, such as TSAT, hemoglobin, total iron-binding capacity, and ferritin. The study identified a significant link (*P* < 0.001) between ID anemia and severe RLS symptoms in adults [46]. While Earley et al. focused on central iron regulation via CSF indicators, Bae et al. showed the systemic impact of iron shortage on RLS, providing a more complete picture of its pathogenesis [11,46]. Both studies emphasize the need to better address iron deficiency at both the systemic and central levels to treat RLS symptoms [11,46].

The remaining two studies in Table 2, by Cielo et al. [42] and Rosen et al. [49], focus on pediatric populations and provide new insights into RLS and ID. Rosen et al. studied juvenile patients with RLS aged 5-18 years. They discovered that changes in serum ferritin levels were related to considerable but nonsignificant symptom alleviation when utilizing oral iron supplementation (ferrous sulfate + vitamin C, *P* > 0.05) [49]. Cielo et al. found that preterm children (aged 5-12 years) had a greater prevalence of RLS, which was associated with low serum ferritin levels (*P* < 0.001) [42]. While Rosen et al. pointed out the limited effectiveness of oral iron in infants with established RLS, Cielo et al. underlined the need for early iron administration in at-risk groups, such as those with a history of prematurity [42,49].

Impact of Iron Supplementation on RLS

The ideal serum ferritin cutoff for diagnosing ID varies with population characteristics; however, recent research suggests <45 μg/L as an effective threshold in elderly patients, with or without anemia, mainly since patients with RLS in this group show a high prevalence of iron deficiency [9]. Current recommendations emphasize evaluating iron status and initiating iron therapy when peripheral iron levels are low [12]. Clinical and scientific research on oral and IV iron therapies for RLS and periodic limb movement disorder (PLMD) has grown substantially in recent years [12].

While readily accessible, oral iron supplementation like ferrous sulfate has shown limited effectiveness due to poor absorption and compliance issues, including gastrointestinal discomfort [53]. Iron absorption is tightly regulated by erythropoiesis, with hepcidin limiting further absorption once iron stores are adequate for red blood cell production [26]. This mechanism reduces the efficacy of oral iron supplementation as serum ferritin levels rise, offering minimal benefit when ferritin exceeds 75-100 μg/L [54]. In contrast, IV iron therapy, particularly FCM, has demonstrated superior efficacy in alleviating RLS symptoms [12]. IV iron is especially effective in patients with serum ferritin levels exceeding 75 μg/L, whereas oral iron provides little benefit [54]. Clinical guidelines recommend IV FCM for adults with moderate-to-severe RLS with serum ferritin levels ≤ 300 μg/L and TSAT below 45% [11]. Notably, FCM is preferred among IV formulations due to its slow-release properties, which reduce the risk of free iron toxicity and allow for larger doses in a single session [55].

Oral iron treatment for three months may reduce sleep disturbances, PLMS, and RLS symptoms in children with RLS and PLMD [12]. However, it is unclear if treatment can be stopped once symptoms improve [12]. IV iron sucrose was safe and effective in children with ID anemia who had been refractory to oral iron [56]. IV iron can be considered without a prior oral iron trial if significant comorbidity will impair iron absorption. Serum ferritin ≥50 μg/L is regarded as an adequate therapeutic target in children [57].

The relationship between serum and CSF ferritin further underscores the potential of IV therapy [12]. Although CSF ferritin levels are generally lower in patients with RLS, they are positively correlated with serum ferritin, albeit with variability [11]. This suggests that raising serum ferritin above 200 μg/L could improve brain iron levels, which oral iron alone may not achieve due to absorption limitations [12]. The historical use of IV iron for RLS dates back to 1953, but it fell out of favor until recent studies on CSF ferritin revived interest [58].

Macher et al. examined the effectiveness of IV versus oral iron therapy in a secondary analysis of the IronWoMan randomized controlled trial [49]. Both therapies significantly improved fatigue and quality of life among iron-deficient blood donors, with IV iron showing slightly more significant benefits in sleep quality and RLS symptom improvement. However, the differences were not statistically significant [49].In summary, while oral iron is a practical first-line option, its limitations in absorption and efficacy at higher ferritin levels make IV iron, particularly FCM, a more sensible alternative for moderate-to-severe RLS. This approach offers a promising path for managing RLS.

Additional Variability in Findings 

Variability in findings regarding iron therapy for RLS reflects differences in formulations, dosages, and outcome measures. For instance, Davis et al. and Lee et al. evaluated oral iron but reported differing outcomes: Davis et al. focused on sleep and quality of life, while Lee et al. assessed symptom severity using the International Restless Legs Scale (IRLS), noting moderate improvements [59,60]. In contrast, Trenkwalder et al. demonstrated the superior efficacy of IV FCM, which significantly increased serum ferritin and TSAT levels compared to placebo, particularly in patients with severe symptoms [27].

The efficacy of IV formulations also varies. Earley et al. assessed iron sucrose and noted limited effectiveness, while Sloand et al. reported extended symptom relief with iron dextran, achieving an average treatment duration of four weeks despite a small sample size [61,62]. Grote et al. and Cho et al. both investigated the effects of IV iron on RLS symptoms, but they reached different conclusions [63,64]. Grote et al. found that iron sucrose did not show superiority over placebo at 11 weeks but provided symptom relief at seven weeks and long-term control over 12 months [64]. In contrast, Cho et al. demonstrated that FCM significantly improved RLS symptoms at six weeks and maintained benefits for up to 30 weeks, reinforcing its efficacy in both short- and long-term treatment [63].

Guidelines reflect that IV iron therapy is promising but still has uncertain potential, particularly with FCM [12]. Randomized trials, including those by Allen et al. and Trenkwalder et al., suggest IV formulations can improve RLS symptoms, but results vary, with Trenkwalder et al. showing delayed efficacy at 12 weeks [27,65]. Open-label studies, such as those by Sloand et al., highlight the short-term benefits of IV iron dextran but emphasize the need for more extensive and rigorous trials to validate long-term relief [62]. Allen et al. (2018) recommend oral iron (ferrous sulfate 325 mg + vitamin C 100 mg) for 12 weeks if serum ferritin is ≤75 μg/L, or IV iron (FCM, 1000 mg over 15 minutes) for ferritin levels between 75 and 100 μg/L or when oral iron is unsuitable, poorly tolerated, or rapid symptom relief is needed [12]. Both therapies should be limited to patients with TSAT <45% to avoid iron overload [12].

Variability in treatment outcomes suggests that while iron deficiency plays a significant role in RLS, it is not the sole factor. O'Keeffe et al. found that iron deficiency contributed to RLS in elderly patients, with symptom improvement depending on baseline ferritin levels [9]. Bang et al. reported that nearly two-thirds of women with iron-deficient RLS continued to experience symptoms despite ferritin normalization, indicating other contributing factors [66]. Similarly, Dosman et al. noted the lack of definitive evidence for iron efficacy in pediatric RLS, highlighting the need for additional management strategies [67]. These findings underscore that complex, multifactorial interactions beyond iron status alone influence RLS.

A meta-analysis by Avni et al. found that iron supplementation significantly improved IRLSS scores and quality of life in patients with RLS, with both oral and IV iron showing efficacy [68]. However, the study emphasized further research to identify which subgroups benefit the most [68]. Elstrott et al. highlighted the expanding use of IV iron beyond IDA treatment, including its potential role in RLS, without directly addressing variations in individual response to iron therapy [69].

Limitations

Understanding the role of iron in the pathophysiology and treatment of RLS remains challenging due to several key limitations in existing research. RLS is thought to stem from dysfunctional iron metabolism in the brain, particularly in areas like the substantia nigra, where iron is crucial for dopamine synthesis and function. However, serum markers such as ferritin and % TSAT, commonly used to assess iron levels, do not accurately reflect brain iron stores. This disconnect makes it difficult to establish links between peripheral ID and the neurological mechanisms underlying RLS.

The variability in defining ID further complicates research. Different studies use inconsistent cutoffs for ferritin and % TSAT, leading to conflicting findings and making it hard to determine who would benefit most from iron-based interventions. Additionally, circadian fluctuations in iron levels, which can worsen RLS symptoms at night, are often overlooked despite their potential relevance to understanding disease progression and treatment timing.

The lack of standardized protocols in treatment studies also poses a problem. Differences in the type of iron used (e.g., FCM vs. iron sucrose), dosage, and duration of therapy lead to inconsistent outcomes, limiting the generalizability of findings. While iron supplementation has shown short-term benefits in some cases, long-term data on the safety and efficacy of repeated treatments, particularly IV iron, are sparse. This is especially concerning for patients with recurrent or chronic RLS symptoms.

Another critical gap lies in the study of high-risk groups, such as children, elderly individuals, and those with comorbidities, who may have unique pathophysiological features. Small sample sizes and publication bias further hinder our understanding by limiting the reliability and breadth of available data.

Future Research Directions 

Future research must tackle several critical gaps to understand iron's role in RLS fully. Studies should focus on brain iron levels and their connection to the dopaminergic system, while also standardizing diagnostic criteria, treatment protocols, and outcome measures. Carefully designed, placebo-controlled trials with standardized dosing are essential to clarify the safety and efficacy of iron therapy across diverse populations. Exploring biomarkers like % TSAT or ferritin-transferrin levels in CSF could provide better insights into how iron influences CNS iron status and predict treatment outcomes. Research should also address high-risk groups, such as pregnant women and preterm infants, to develop tailored recommendations for these populations. Additionally, investigating how circadian rhythms influence serum and brain iron levels could lead to personalized treatment strategies that align with patients' symptom patterns, ultimately advancing our understanding of RLS pathophysiology and improving therapeutic outcomes.

## Conclusions

RLS arises from brain ID and dopaminergic dysfunction, with iron dysregulation in areas like the substantia nigra playing a central role. Reduced brain iron impairs dopamine metabolism, driving the sensory-motor symptoms of RLS. Management strategies focus on correcting iron deficiency with IV iron supplementation, particularly FCM, showing efficacy in severe cases and those unresponsive to oral iron. Variability in patient responses highlights the need for individualized treatment plans and standardized protocols. Advancing research into the pathophysiology and tailored management approaches will be critical for improving outcomes in RLS care.
